# Supplementary material for: Oral reading promotes predictive processing in Chinese sentence reading: eye movement evidence
Source: PeerJ. 2024 Oct 17;12:e18307. doi: 10.7717/peerj.18307 (PMC11491060; doi:10.7717/peerj.18307)
Supplement: Supplemental Information 2 [file peerj-12-18307-s002.docx]

***Section1 Protocol for power analysis (S1)***

I did the power analysis via PANGEA (<https://jakewestfall.shinyapps.io/pangea/>)

**Step 1: Specify the design**

Given that we adopted a counter-balanced design, we selected the ‘6. Participants crossed w/ random Stimuli, counter-balanced (Kenny & Smith, 1980). ’

- **Fixed Factors:**
- Reading Mode: 2 levels (Aloud, Silent)
- Cloze Value: 2 levels (High, Low) (The term "Cloze value" is used to represent word predictability to avoid repetition with "Participant," both of which are labeled as P.)
- **Random Factors:**
- Participant: 16 levels per group
- Stimulus: 22 levels per treatment or condition
- **Design:**
  Participants are nested within groups, stimuli are nested within reading mode, and stimuli are nested within cloze value (i.e., predictability). Participants are crossed with stimuli.
- **Replicates:**
  Each participant reads each sentence only once (Replicates = 1).

Tabel S1 Grouping Overview

| Group | Participant | S1-S22 | S23-S44 | S45-S66 | S67-S88 |
| --- | --- | --- | --- | --- | --- |
| G1 | P1-P16 | AH | AL | SH | SL |
| G2 | P17-P32 | AL | SH | SL | AH |
| G3 | P33-P48 | SH | SL | AH | AL |
| G4 | P48-P64 | SL | AH | AL | SH |


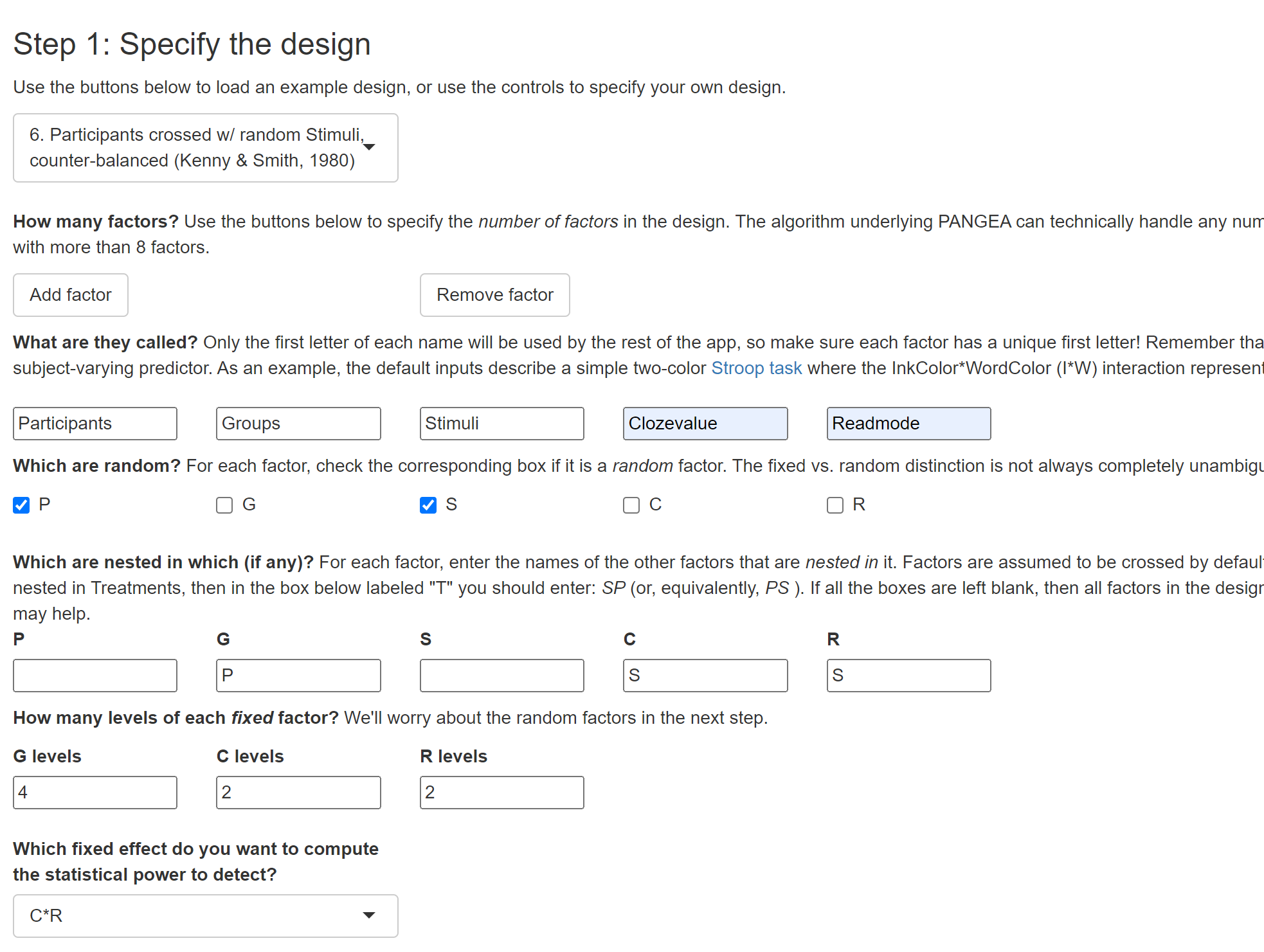


**Step 2: Specify the Effect Size**

- The default effect size suggested by PANGEA is *d*=0.45, based on a distribution of Cohen’s d values derived from a meta-analysis by Richard, Bond Jr., & Stokes-Zoota (2003). Alternatively, effect sizes can be adjusted to other values as needed.
- Each participant reads one sentence or stimulus only once, so the replicates (observations per Participant × Stimulus) equal 1.
- There are 16 participants in each group of our current experiment.
- Each participant reads 22 stimuli per Reading Mode × Cloze Value combination (as shown in the table above), thus Number of S’s (per C*R)=22
- Finally, the values of Variance Partitioning Coefficients (VPCs) are set to the default values suggested by PANGEA, based on the hierarchical ordering principle (We do not need to enter these values).

If we adopt the default effect size of *d*=0.45 suggested by PANGEA, the power of the current design is 0.989, which exceeds the recommended threshold of 0.8.


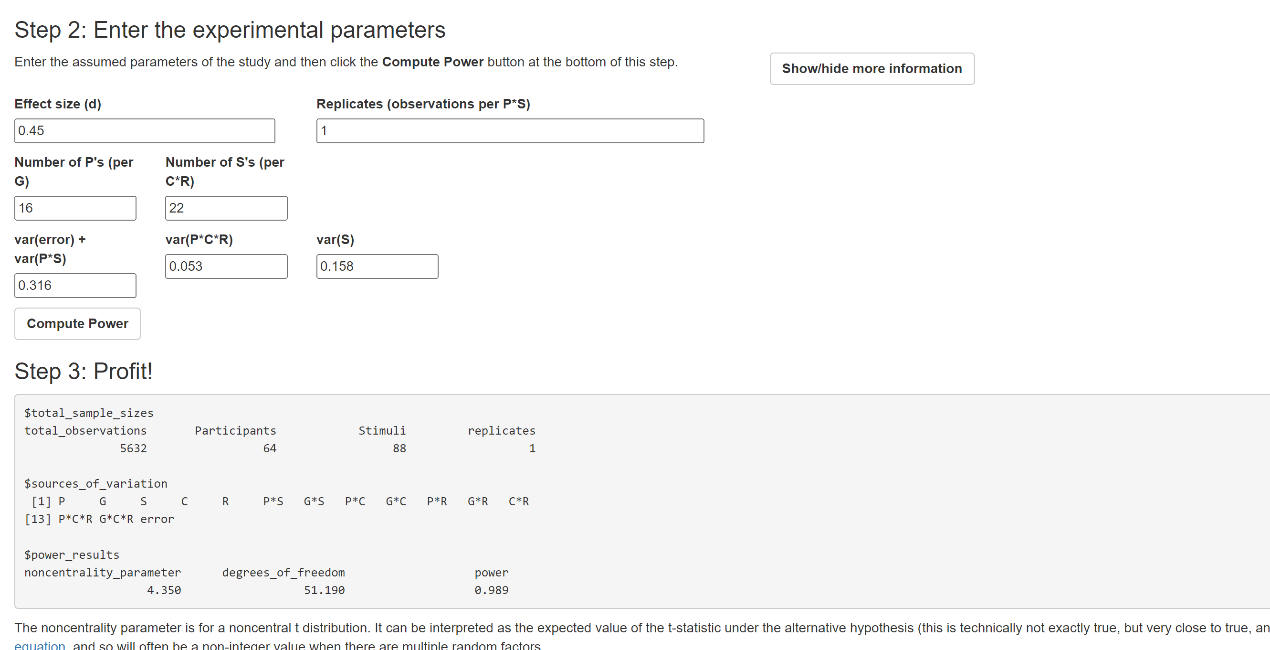


When setting the effect size to 0.4, which is considered typical in psychology (Brysbaert & Stevens, 2018; Kühberger, Fritz, & Scherndl, 2014; Open Science Collaboration, 2015), or to 0.3, the resulting power analysis is as follows: The results indicate that we need between 20 and 64 participants to achieve greater than 80% statistical power for detecting an effect size within the range of *d*=[0.3,0.4].


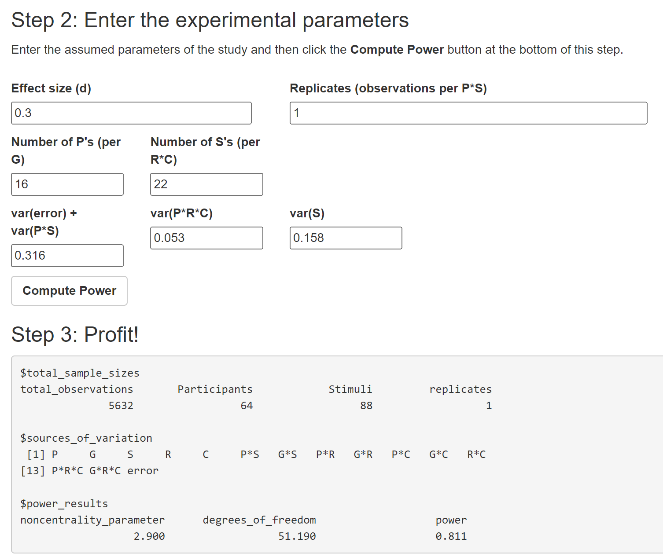

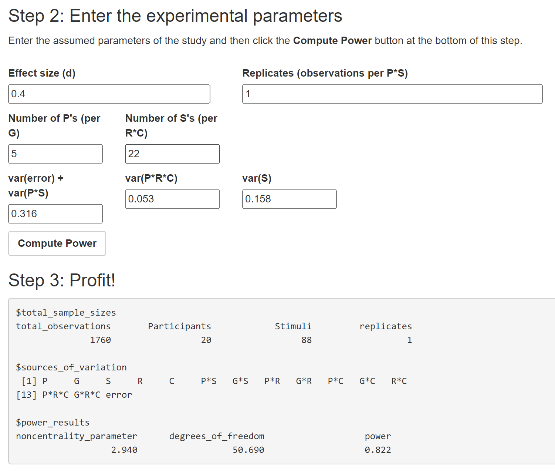


***Section 2 Explanation of the experiment procedure (S2)***

Tabel S1 Grouping Overview

| Group/block | Participant | S1-S22 | S23-S44 | S45-S66 | S67-S88 |
| --- | --- | --- | --- | --- | --- |
| G1 | P1-P16 | AH | AL | SH | SL |
| G2 | P17-P32 | AL | SH | SL | AH |
| G3 | P33-P48 | SH | SL | AH | AL |
| G4 | P48-P64 | SL | AH | AL | SH |

Note: A represents oral reading while S represents silent reading. H represents High predictable words while L represents Low predictable words.


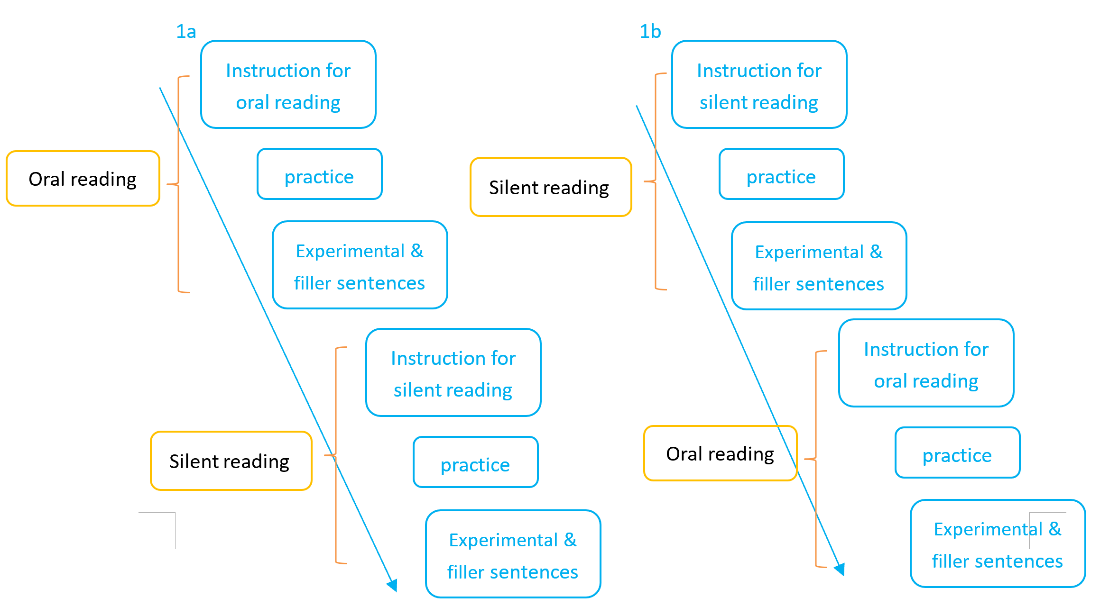


Figure S1. Experimental Procedure

Our experiment utilized a counter-balanced design consisting of four blocks or groups, as illustrated in the above table. For blocks 1&2, readers read sentences orally first, followed by a rest screen instructing silent reading, then five practice sentences to adapt to the new reading mode (Figure S1a). For blocks 3&4, readers read sentences silently first, followed by a rest screen instructing read aloud, then five practice sentences for adapting to reading aloud (Figure 1Sb).

***Section 3 Results of Mixed-Effects Models for Reading Mode, and Predictability(S3)***

The results based on the raw data are reported in Table S3, while those based on the log-transformed data are reported in Table S4.

Table S3 Results of Mixed-Effects Models for Reading Mode, and Predictability (raw data)

| measures | Fixed factor | *b* | CI | *SE* | *t/z* |
| --- | --- | --- | --- | --- | --- |
| Skip | Intercept | -1.34 | [-1.54, -1.15] | .1 | -13.6 |
|  | Reading mode | -1.92 | [-2.07, -1.77] | .08 | **-24.79*** |
|  | Predictability | -.18 | [-.33, -.03] | .07 | **-2.43*** |
|  | Readingmode×Predictability | -.09 | [-.38, .21] | .15 | -.59 |
| FFD | Intercept | 253 | [245, 262] | 4 | 58.15 |
|  | Reading mode | 62 | [53, 72] | 5 | **13.63*** |
|  | Predictability | 8 | [2, 14] | 3 | **2.48*** |
|  | Readingmode×Predictability | 17 | [5, 29] | 6 | **2.74*** |
| SFD | Intercept | 256 | [248, 265] | 4 | 57.09 |
|  | Reading mode | 71 | [61, 80] | 5 | **14.79*** |
|  | Predictability | 9 | [3, 16] | 3 | **2.85*** |
|  | Readingmode×Predictability | 22 | [9, 35] | 7 | **3.35*** |
| GD | Intercept | 295 | [285, 305] | 5 | 57.18 |
|  | Reading mode | 119 | [105, 133] | 7 | **16.68*** |
|  | Predictability | 20 | [10, 29] | 5 | **4.18*** |
|  | Readingmode×Predictability | 34 | [17, 52] | 9 | **3.80*** |
| TRT | Intercept | 352 | [337, 367] | 8 | 45.66 |
|  | Reading mode | 121 | [103, 138] | 9 | **13.69*** |
|  | Predictability | 28 | [18, 39] | 6 | **5.16*** |
|  | Readingmode×Predictability | 22 | [0, 44] | 11 | **1.99*** |
| RPD | Intercept | 342 | [327, 356] | 7 | 47.63 |
|  | Reading mode | 123 | [101, 145] | 11 | **11.12*** |
|  | Predictability | 18 | [4, 32] | 7 | **2.55*** |
|  | Readingmode×Predictability | 34 | [6, 62] | 14 | **2.37*** |

Note. *Asterisks indicate significant effects where *t/z* > 1.96, *p* < .05. CI = 95% Confidence Interval. FFD: first fixation duration. SFD: single fixation duration. GD: gaze duration. TRT: total reading time. RPD: regression path duration. The initial attempt to include the maximum random-effect structures (depvar~readingmode*predictability+(1+readingmode*predictability|pp)+(1+readingmode*predictability|item)) resulted in convergence failure (convergence code 0), To address this, the model was incrementally simplified, beginning with the random structure for stimuli. First, the random effect correlations were removed, followed by the random slopes. If the model still failed to converge, the random structure for subjects was similarly reduced by removing correlations and then slope until convergence was achieved.

The final models that successfully converged were as follows:

for skipping rate: depvar.glmer1=glmer(depvar~readingmode*predictability+ (1|pp)+(1|item),datafile, family=binomial);

for FFD, SFD, and RPD: model= lmer(depvar ~readingmode*predictability+ (1+readingmode|pp) + (1|item), datafile);

for GD and TRT: model=lmer(depvar~readingmode*predictability+ (1+readingmode+predictability|pp)+(1|item), datafile).

.

Table S4 Results of Mixed-Effects Models for Reading Mode, and Predictability (The continuous variables were log-transformed)

| measures | Fixed factor | *b* | CI | *SE* | *t/z* |
| --- | --- | --- | --- | --- | --- |
| FFD | Intercept | 5.46 | [5.43, 5.49] | .02 | 339.86 |
|  | Reading mode | .23 | [.19, .26] | .02 | **12.45 *** |
|  | Predictability | .02 | [, .05] | .01 | **2.13 *** |
|  | Readingmode×Predictability | .05 | [.01, .1] | .02 | **2.35 *** |
| SFD | Intercept | 5.47 | [5.44, 5.5] | .02 | 336.26 |
|  | Reading mode | .26 | [.22, .29] | .02 | **14.06 *** |
|  | Predictability | .03 | [.01, .05] | .01 | **2.51 *** |
|  | Readingmode×Predictability | .07 | [.03, .12] | .02 | **3.08 *** |
| GD | Intercept | 5.58 | [5.54, 5.61] | .02 | 332.04 |
|  | Reading mode | .38 | [.33, .42] | .02 | **16.58 *** |
|  | Predictability | .05 | [.03, .08] | .01 | **4.08 *** |
|  | Readingmode×Predictability | .09 | [.04, .14] | .03 | **3.38 *** |
| TRT | Intercept | 5.72 | [5.68, 5.76] | .02 | 288.21 |
|  | Reading mode | .35 | [.3, .39] | .02 | **14.64 *** |
|  | Predictability | .08 | [.05, .11] | .01 | **5.47 *** |
|  | Readingmode×Predictability | .03 | [-.02, .09] | .03 | 1.15 |
| RPD | Intercept | 5.68 | [5.64, 5.71] | .02 | 301.26 |
|  | Reading mode | .36 | [.31, .42] | .03 | **12.66 *** |
|  | Predictability | .05 | [.02, .08] | .02 | **3.14 *** |
|  | Readingmode×Predictability | .08 | [.03, .14] | .03 | **2.79 *** |

Note. *Asterisks indicate significant effects where *t/z* > 1.96, *p* < .05. CI = 95% Confidence Interval. FFD: first fixation duration. SFD: single fixation duration. GD: gaze duration. TRT: total reading time. RPD: regression path duration. The final models that successfully converged were as follows:

for FFD, SFD, GD: model= lmer(depvar ~readingmode*predictability+ (1+readingmode|pp) + (1|item), datafile);

for RPD and TRT: model=lmer(depvar~readingmode*predictability+ (1+readingmode+predictability|pp)+(1|item), datafile).

***Section 4 Results of Mixed-Effects Models for Group, Reading Mode, and Predictability (S4)***

Table S5 Results of Mixed-Effects Models for Group, Reading Mode, and Predictability

| measures | Fixed factor | *b* | CI | *SE* | *t/z* |
| --- | --- | --- | --- | --- | --- |
| Skip | (Intercept) | -1.32 | [-1.49, -1.15] | .08 | -15.6 |
|  | group2-1 | .68 | [.36, 1.02] | .17 | **4.12*** |
|  | readingmode2-1 | -1.88 | [-2.03, -1.73] | .08 | **-24.29*** |
|  | predictability2-1 | -.18 | [-.33, -.03] | .08 | **-2.43*** |
|  | group2-1:readingmode2-1 | -.52 | [-.82, -.22] | .15 | **-3.42*** |
|  | group2-1:predictability2-1 | -.05 | [-.35, .25] | .15 | -.33 |
|  | readingmode2-1:predictability2-1 | -.1 | [-.39, .2] | .15 | -.63 |
|  | group2-1:readingmode2-1:predictability2-1 | .28 | [-.31, .88] | .3 | .94 |
| FFD | (Intercept) | 253 | [244, 261] | 4 | 58.64 |
|  | group2-1 | -12 | [-29, 4] | 8 | -1.45 |
|  | readingmode2-1 | 64 | [55, 72] | 4 | **14.44*** |
|  | predictability2-1 | 8 | [2, 14] | 3 | **2.6*** |
|  | group2-1:readingmode2-1 | 25 | [7, 42] | 9 | **2.8*** |
|  | group2-1:predictability2-1 | 3 | [-9, 15] | 6 | .51 |
|  | readingmode2-1:predictability2-1 | 16 | [4, 28] | 6 | **2.55*** |
|  | group2-1:readingmode2-1:predictability2-1 | -5 | [-29, 20] | 12 | -.38 |
| SFD | (Intercept) | 256 | [247, 265] | 4 | 57.85 |
|  | group2-1 | -14 | [-31, 3] | 9 | -1.62 |
|  | readingmode2-1 | 72 | [63, 81] | 5 | **15.18*** |
|  | predictability2-1 | 10 | [4, 17] | 3 | **3.01*** |
|  | group2-1:readingmode2-1 | 20 | [2, 39] | 9 | **2.12*** |
|  | group2-1:predictability2-1 | 4 | [-9, 17] | 7 | .65 |
|  | readingmode2-1:predictability2-1 | 21 | [8, 34] | 7 | **3.13*** |
|  | group2-1:readingmode2-1:predictability2-1 | -7 | [-34, 19] | 13 | -.55 |
| GD | (Intercept) | 294 | [285, 304] | 5 | 60.01 |
|  | group2-1 | -26 | [-44, -8] | 9 | **-2.85*** |
|  | readingmode2-1 | 121 | [107, 135] | 7 | **16.87*** |
|  | predictability2-1 | 20 | [10, 29] | 5 | **4.12*** |
|  | group2-1:readingmode2-1 | 24 | [-4, 52] | 14 | 1.67 |
|  | group2-1:predictability2-1 | -8 | [-27, 11] | 10 | -.84 |
|  | readingmode2-1:predictability2-1 | 34 | [16, 52] | 9 | **3.75*** |
|  | group2-1:readingmode2-1:predictability2-1 | 3 | [-33, 39] | 18 | .14 |
| TRT | (Intercept) | 351 | [337, 365] | 7 | 49.59 |
|  | group2-1 | -51 | [-76, -26] | 13 | **-3.98*** |
|  | readingmode2-1 | 122 | [105, 140] | 9 | **13.77*** |
|  | predictability2-1 | 29 | [17, 40] | 6 | **4.96*** |
|  | group2-1:readingmode2-1 | 23 | [-12, 58] | 18 | 1.3 |
|  | group2-1:predictability2-1 | -12 | [-34, 11] | 12 | -1.01 |
|  | readingmode2-1:predictability2-1 | 21 | [-1, 43] | 11 | **1.91+** |
|  | group2-1:readingmode2-1:predictability2-1 | -12 | [-55, 32] | 22 | -0.52 |
| RPD | (Intercept) | 340 | [327, 353] | 7 | 50.22 |
|  | group2-1 | -39 | [-63, -16] | 12 | **-3.27*** |
|  | readingmode2-1 | 127 | [105, 148] | 11 | **11.37*** |
|  | predictability2-1 | 18 | [4, 32] | 7 | **2.5*** |
|  | group2-1:readingmode2-1 | 29 | [-14, 73] | 22 | 1.32 |
|  | group2-1:predictability2-1 | -15 | [-43, 14] | 14 | -1.03 |
|  | readingmode2-1:predictability2-1 | 34 | [5, 62] | 14 | **2.32*** |
|  | group2-1:readingmode2-1:predictability2-1 | -10 | [-67, 47] | 29 | -0.35 |

Note. *Asterisks indicate significant effects where *t/z* > 1.96, *p* <. 05. CI = 95% Confidence Interval. FFD: first fixation duration. SFD: single fixation duration. GD: gaze duration. TRT: total reading time. RPD: regression path duration. The median reading speed was computed separately for each participant within each block. Participants in each block were then categorized into fast and slow reading groups based on the respective median speeds. Thus, this categorization was carried out separately for oral and silent reading modes. The final models that successfully converged were as follows:

For skipping rate: model_SKIP=glmer(depvar ~ group*readingmode*predictability + (1|pp)+ (1|item), datafile, family=binomial, control=glmerControl(optimizer="Nelder_Mead",optCtrl = list(maxfun = 200000)));

for FFD, SFD, RPD: model= lmer(depvar ~ group*readingmode*predictability+ (1+readingmode|pp) + (1|item), datafile);

for GD and TRT: model=lmer(depvar~ group*readingmode*predictability+ (1+readingmode+predictability|pp)+(1|item), datafile).

***Section 5 ANCOVA Analysis Incorporating Reading Speed as a Continuous Variable (S5)***

Table S6 ANCOVA Analysis Incorporating Reading Speed as a Continuous Variable

| measures | Fixed factor | *b* | CI | *SE* | *t/z* |
| --- | --- | --- | --- | --- | --- |
| Skip | Intercept | -1.33 | [-1.49, -1.16] | .08 | -16.09 |
|  | Reading mode | -1.3 | [-1.52, -1.09] | .11 | **-11.74*** |
|  | Predictability | -.17 | [-.31, -.02] | .07 | **-2.22*** |
|  | Reading speed | .39 | [.29, .49] | .05 | **7.36*** |
|  | Readingmode×Predictability | -.11 | [-.4, .19] | .15 | -.71 |
| FFD | Intercept | 253 | [245, 261] | 4.18 | 60.5 |
|  | Reading mode | 38 | [26, 49] | 5.82 | **6.47*** |
|  | Predictability | 7 | [1, 13] | 3.05 | **2.43*** |
|  | Reading speed | -19 | [-25, -13] | 3.03 | **-6.25*** |
|  | Readingmode×Predictability | 17 | [5, 29] | 6.1 | **2.77*** |
| SFD | Intercept | 256 | [248, 264] | 4.3 | 59.59 |
|  | Reading mode | 46 | [34, 58] | 6.14 | **7.57*** |
|  | Predictability | 9 | [3, 16] | 3.31 | **2.83*** |
|  | Reading speed | -18 | [-24, -12] | 3.11 | **-5.9*** |
|  | Readingmode×Predictability | 22 | [9, 35] | 6.61 | **3.36*** |
| GD | Intercept | 294 | [285, 303] | 4.58 | 64.11 |
|  | Reading mode | 77 | [60, 94] | 8.69 | **8.89*** |
|  | Predictability | 20 | [11, 29] | 4.51 | **4.48*** |
|  | Reading speed | -32 | [-41, -24] | 4.29 | **-7.53*** |
|  | Readingmode×Predictability | 34 | [16, 52] | 9.01 | **3.77*** |
| TRT | Intercept | 350 | [337, 362] | 6.36 | 54.99 |
|  | Reading mode | 21 | [0, 42] | 10.52 | **2.03*** |
|  | Predictability | 28 | [16, 39] | 5.67 | **4.86*** |
|  | Reading speed | -75 | [-85, -65] | 4.88 | **-15.45*** |
|  | Readingmode×Predictability | 22 | [1, 43] | 10.8 | **2.05*** |
| RPD | Intercept | 338 | [326, 351] | 6.35 | 53.28 |
|  | Reading mode | 48 | [21, 74] | 13.16 | **3.62*** |
|  | Predictability | 18 | [4, 32] | 7.08 | **2.52*** |
|  | Reading speed | -60 | [-73, -47] | 6.57 | **-9.19*** |
|  | Readingmode×Predictability | 34 | [6, 61] | 14.16 | **2.37*** |

Note. *Asterisks indicate significant effects where *t/z* > 1.96, *p* <. 05. CI = 95% Confidence Interval. FFD: first fixation duration. SFD: single fixation duration. GD: gaze duration. TRT: total reading time. RPD: regression path duration. The final models that successfully converged were as follows:

For skipping rate: glmer(depvar~readingmode*predictability+speed + (1|pp)+ (1|item), datafile, family=binomial, control=glmerControl(optimizer="Nelder_Mead",optCtrl = list(maxfun = 200000)));

For FFD, SFD, GD, RPD: lmer(depvar ~readingmode*predictability+ speed+(1+readingmode|pp) + (1|item), datafile);

For TRT: lmer(depvar ~readingmode*predictability+ speed+(1+readingmode+ predictability|pp) + (1|item), datafile).
